# Multicomponent perioperative interventions to improve outcomes for frail patients: a systematic review

**DOI:** 10.1186/s12877-024-04985-4

**Published:** 2024-04-26

**Authors:** Vivian Ka-Yan Kwok, Natasha Reid, Ruth E Hubbard, Harshithaa Thavarajah, Emily H Gordon

**Affiliations:** 1 Princess Alexandra Hospital, Metro South Hospital and Health Service, Brisbane, QLD Australia; 2 Centre for Health Services Research, The University of Queensland, Brisbane, Australia; 3 Toowoomba Hospital, Darling Downs Health, Toowoomba, QLD Australia

**Keywords:** Frail elderly, Perioperative interventions, Postoperative outcomes, Multicomponent

## Abstract

**Background:**

Preoperative frailty is associated with increased risk of adverse outcomes. In 2017, McIsaac and colleagues’ systematic review found that few interventions improved outcomes in this population and evidence was low-quality. We aimed to systematically review the evidence for multicomponent perioperative interventions in frail patients that has emerged since McIsaac et al.’s review.

**Methods:**

PUBMED, EMBASE, Cochrane, and CINAHL databases were searched for English-language studies published since January 1, 2016, that evaluated multicomponent perioperative interventions in patients identified as frail. Quality was assessed using the National Institute of Health Quality Assessment Tool. A narrative synthesis of the extracted data was conducted.

**Results:**

Of 2835 articles screened, five studies were included, all of which were conducted in elective oncologic gastrointestinal surgical populations. Four hundred and thirteen patients were included across the five studies and the mean/median age ranged from 70.1 to 87.0 years. Multicomponent interventions were all applied in the preoperative period. Two studies also applied interventions postoperatively. All interventions addressed exercise and nutritional domains with variability in timing, delivery, and adherence. Multicomponent interventions were associated with reduced postoperative complications, functional deterioration, length of stay, and mortality. Four studies reported on patient-centred outcomes. The quality of evidence was fair.

**Conclusions:**

This systematic review provides evidence that frail surgical patients undergoing elective oncologic gastrointestinal surgery may benefit from targeted multicomponent perioperative interventions. Yet methodological issues and substantial heterogeneity of the interventions precludes drawing clear conclusions regarding the optimal model of care. Larger, low risk of bias studies are needed to evaluate optimal intervention delivery, effectiveness in other populations, implementation in health care settings and ascertain outcomes of importance for frail patients and their carers.

**Supplementary Information:**

The online version contains supplementary material available at 10.1186/s12877-024-04985-4.

## Background

The ageing population, along with advances in anaesthetic and surgical techniques, will lead to an increasing number of frail older patients undergoing surgical interventions. Preoperative frailty is associated with increased risk of adverse outcomes. This was objectively quantified in the first study of frailty and surgical outcomes by Mackary and colleagues in 2010, which demonstrated the association of preoperative frailty with increased risk of postoperative complications, increased length of stay (LOS), and discharge to institutional care [1]. Since then, there has been a surge in literature on the impact of frailty on perioperative outcomes [2]. Not only is frailty consistently associated with risk of major morbidity, mortality and readmissions [2–5], it is also associated with new patient-reported disability [6], institutional care, functional decline, and lower quality of life post-surgery [2, 4].

Despite the strong evidence that preoperative frailty in surgical patients results in poor postoperative outcomes, there is limited evidence to date supporting interventions in frail surgical patients. A 2017 systematic review by McIsaac et al. [7] found that few interventions improved outcomes in this patient population. Five of the 11 included studies tested multicomponent interventions and these studies failed to consistently demonstrate improvements in outcomes and most were at high risk of bias [7]. We aimed to systematically review the evidence for multicomponent perioperative interventions in frail patients that has emerged since McIsaac et al.’s [7] review.

## Methods

### Protocol and registration

The protocol for this systematic review was registered with PROSPERO (CRD42021282937) and conducted according to the Preferred Reporting Items for Systematic Reviews and Meta-analyses (PRISMA) reporting guidelines [8].

### Search strategy

We searched PUBMED, EMBASE, Cochrane Central Register of Controlled Trials, CINAHL online databases, with publication dates from January 1, 2016 to October 20, 2021, with updated searches on August 27, 2022 and March 29, 2023. The search terms combined Medical Subject Headings (MeSH) and free text words (See Supplementary for full search strategy). Publications were limited to English language. Additional publications were identified by searching reference lists of included papers.

### Study selection

Two reviewers, VK and HT, in the initial database search and, VK and NR or VK and EG in the updated searches, independently screened titles, abstracts and full texts. Reasons for exclusion were documented. Discrepancies on whether a study met inclusion criteria were resolved by discussion and consensus.

Inclusion and exclusion criteria are outlined in Table 1. Included studies were randomised controlled trials or quasi-experimental studies of perioperative multicomponent interventions in frail surgical patients aged 18 years and over. Studies were to use a valid frailty measurement tool. This was defined as a composite measure of deficits in two or more health domains. Studies using a single domain measure (such as a physical performance test) were therefore excluded.

**Table 1:** Inclusion and exclusion criteria

	Inclusion Criteria	Exclusion Criteria
Population	Aged ≥ 18 years Underwent surgery (all surgical settings including elective/emergency/major or minor surgeries or procedures) Used a valid frailty measure and majority of the sample classified as frail.	Aged < 18 years Did not undergo surgery Did not use a valid frailty measure. Used a valid frailty measure but majority of the sample not classified as frail or data for frail group unable to be extracted.
Intervention	Perioperative multicomponent intervention	Interventions targeting a single health domain Interventions forming part of established standard of care protocols, such as ERAS.
Comparator	Standard/usual care Alternative intervention (superiority trial)	
Outcome	Examined relationship between intervention/comparator and one or more outcome(s)	
Study design	Randomised controlled trials, quasi-experimental studies	Observational studies
Publication Criteria	Published and “in press” articles reporting original research results	Conference abstract only, reviews, book chapters, editorials, theses Full text not available
Language	Studies written in English	

Note: ERAS, Enhanced Recovery After Surgery

Perioperative multicomponent interventions were defined as interventions directly related to the patient having or having had surgery that addressed at least two health domains and/or involved two or more healthcare disciplines. Studies evaluating established standard of care protocols only, such as Enhanced Recovery After Surgery (ERAS) protocols, were excluded. There were no inclusion or exclusion criteria relating to the type of study outcomes.

### Data extraction and analysis

Data extraction was conducted by VK and verified by NR and EG using pre-specified data fields as agreed upon by all reviewers. Data included country, study design, sample size and characteristics (age, sex), type of surgery, frailty measure, intervention details (description, timing during perioperative period, setting), and overall study outcomes. Due to the heterogeneity of study designs, interventions and outcomes, a formal meta-analysis was not possible. A narrative synthesis of the results was conducted.

### Assessment of risk of bias

Risk of bias assessments were conducted for all studies using the National Institute of Health Quality Assessment Tool [9] by VK or NR and verified by EG.

## Results

**Fig. 1:**
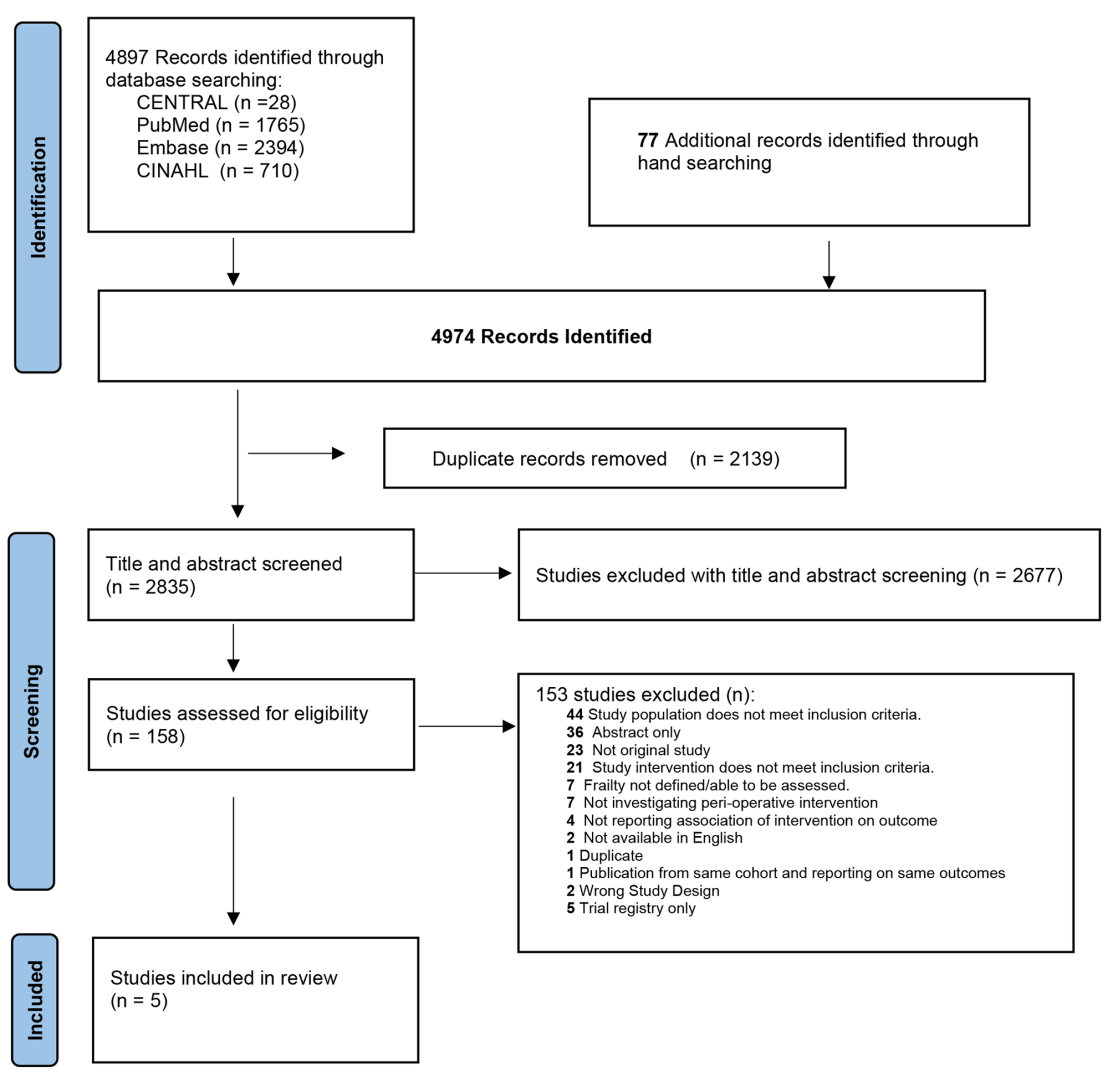
PRISMA diagram of study selection

We identified a total of 4974 articles (Fig. 1). From these, 2139 duplicate articles were removed. Following title, abstract, and full text reviews, five studies were included in the final analysis.

### Study characteristics

Study characteristics are summarised in Table 2. The five studies were conducted in four different countries, including Canada [10], Norway [11], Italy [12], and Japan [13, 14]. Of the five articles included, there were two RCTs [10, 11] and three quasi-experimental studies [12–14]. All five studies recruited patients undergoing elective oncologic gastrointestinal surgery. Specifically, three of the five studies were in colorectal surgery [10, 11, 14] and two studies were in upper gastrointestinal surgery (oesophageal, pancreatic [12] and gastric surgery [12, 13]).

**Table 2:** Characteristics of included studies

First Author, Year of Publication Country	Study Design	Study Population	Mean/Median Age (SD/IQR) in years	% Female	Frailty Measurement Tool	Intervention	Comparator	Outcomes
Carli et al., 2020 [10] Canada	RCT	110 participants (55 = intervention group) Elective colorectal cancer surgery	Intervention: 78 (72–82) Comparator: 82 (75–84)	Intervention: 47.3% Comparator: 58.2%	Fried Phenotype	Multimodal prehabilitation program Embedded within an enhanced recovery pathway (ERAS)	Identical program commenced post-operatively (on discharge from hospital)	Complications 30-days postoperatively (‘comprehensive index’, overall & severe complication rates) LOS ED visits and hospital readmissions 30-days postoperatively Walking capacity, self-reported health status, anxiety and depression, self-reported energy expenditure
Mazzola et al., 2017 [12] Italy	Quasi-Experimental	76 participants (41 = intervention group) Elective curative upper GI oncologic surgery (oesophageal, gastric, pancreatic head)	Intervention: 75 (44–90) Comparator: 75 (59–91)	Intervention: 34% Comparator: 34%	Modified Frailty Index	Multidisciplinary preoperative management plan	“No preoperative treatment, in terms of prehabilitation, had been administered” (p.3)	Mortality 30-days and 3-months postoperatively Overall and severe complication rates LOS Readmission Post-discharge institutionalisation
Ommundsen et al., 2018 [11] Norway	RCT	116 participants (53 = intervention group) Elective colorectal cancer surgery	Intervention: 78.2 (7.4) Comparator: 78.8 (7.8)	Intervention: 58% Comparator: 41%	Vulnerable Elders Survey	Preoperative geriatric assessment and tailored intervention based on the results of the assessment Embedded within an ERAS model	Standard care (ERAS model)	Complications 30-days postoperatively Reoperations and readmissions 30-days postoperatively LOS Mortality 30-days and 3-months postoperatively Discharge status
Suzuki et al., 2022 [14] Japan	Quasi-Experimental	53 participants (15 = intervention group) Elective colorectal cancer surgery	Intervention: 87 (84–88) Comparator: 84 (81–86)	Intervention: 60.0% Comparator: 44.7%	Modified Frailty Index 11	Perioperative Management Team intervention Embedded within an ERAS model	Standard care (ERAS model)	Postoperative high-grade complications Postoperative LOS Reoperations 30- days postoperatively ADL performance Discharge status
Wada et al., 2022 [13] Japan	Quasi-Experimental	58 participants (15 = intervention group) Elective gastric cancer surgery	Intervention: 72.9 (2.5) Comparator: 70.1 (1.7)	Intervention: 33% Comparator: 30%	Clinical Frailty Scale	Nutrition and Exercise Intervention	Standard care (not otherwise specified)	Postoperative complications LOS Neutrophil lymphocyte ratio Lymphocyte to CRP ratio BMI Mean lean mass and mean skeletal muscle mass

Note: RCT, randomised controlled trial; LOS, length of stay; ED, emergency department; ERAS, Enhanced Recovery After Surgery; ADL, activities of daily living; CRP, C-reactive protein; BMI, body mass index

The five studies included a total of 413 participants (range = 58–116), with a mean/median participant age ranging from 70.1 [13] to 87.0 [14] years. The proportion of females ranged from 33% [13] to 60% [14]. Each study used a different frailty measurement tool: Fried Frailty Phenotype [10], Modified Frailty Index (mFI) [12], Vulnerable Elders Survey (VES-13) [11], Modified Frailty Index-11 (mFI-11) [14], and Clinical Frailty Scale (CFS) [13]. One hundred and seventy-nine (43.3%) participants were allocated to an intervention group and 234 (56.7%) were allocated to a comparator group.

### Multicomponent interventions

The interventions are described in Table 3. In all five studies, interventions occurred in the preoperative period. In two of the five studies, interventions also occurred in the postoperative period [11, 14]. In three studies, the interventions were embedded in a well-established ERAS protocol [10, 11, 14]. In four studies [10–13], the interventions included unsupervised home-based programs. Three of these interventions were supplemented by supervised outpatient clinic and inpatient programs [10–12]. In one study [14], the intervention was administered entirely as a supervised inpatient program.

**Table 3:** Perioperative multicomponent interventions: Timing, setting, supervision, domains and personnel

	Timing of Intervention	Setting	Supervision	Health Domains											Personnel								
				Cognition	Comorbidity	ADLs	Medications	Nutrition	Physical Activity	Psychological Health	Smoking Cessation	Respiratory Function	Swallow Function	Oral Health	Geriatrician	Kinesiologist/physical Therapist	Nutritionist/Dietitian	Nurse	Surgeon	Anaesthetist	Dental surgeon or hygienist	Pharmacist	Speech Pathologist
Carli et al. [10]	Preoperatively for 4 weeks	Home + Outpatient Clinic	Unsupervised home-based program + Supervised exercise sessions					✔	✔	✔	✔					✔	✔	✔					
Mazzola et al. [12]	Preoperatively (5 days to 2 weeks)	Home +- Inpatient Nutrition Unit	Unsupervised home-based program +- Supervised NJ/PN					✔	✔		✔	✔					✔	✔	✔	✔			
Ommundsen et al. [11]	Preoperatively (GA completed median 6 days preoperatively) Postoperatively as required	Home +- Inpatient Surgical Unit	Unsupervised home-based program +- Supervised postoperative complication prevention	(✔)	(✔)		(✔)	(✔)	(✔)			(✔)			✔	(✔)		(✔)					
Suzuki et al. [14]	Preoperatively for those admitted ≥ 1 week before surgery Postoperatively for all	Inpatient Surgical Unit	Supervised	✔		✔		✔	✔				✔	✔		✔	✔	✔	✔	✔	✔	✔	✔
Wada et al. [13]	Preoperatively (median 13 days)	Home	Unsupervised home-based program					✔	✔							✔	✔						

Note: NJ, nasojejunal; PN, parenteral nutrition; ADLs, activities of daily living

Suzuki and colleagues’ [14] study was by far the most comprehensive, addressing multiple domains in the pre- and postoperative periods in all patients. In the study by Ommundsen et al. [11], the pre- and postoperative intervention was individualised based upon findings from a geriatric assessment. Consequently, the health domains addressed by the intervention varied for each patient and, in some cases (*N* = 9) no interventions were prescribed. The most commonly addressed health domains in all studies were physical activity and nutrition.

Physical activity programs varied in terms of the type of exercise prescribed (e.g., aerobic [10], resistance training [10, 13], stretching [10], functional retraining [14]), location (e.g., clinic-based [10], home-based [10–13], inpatient nutrition unit [12], inpatient surgical unit [11, 14]), intensity (e.g., weekly [10] versus thrice-weekly [12]), duration of the program (e.g., four weeks [10] versus < 6 days [11]) and supervision by health professionals [14]. Two studies specified involvement of a physical activity specialist [10, 14] and one involved a full rehabilitation team [13].

Similarly, nutritional interventions varied in terms of what nutritional support was prescribed. All included nutritional counselling regarding protein and caloric intake and most included protein supplementation [10–12, 14]. Timing and duration of the nutritional intervention ranged from five days [12] to four weeks [10] preceding surgery. Three studies were prepared to admit patients for enteral or parenteral nutrition [11, 12, 14]. In one study, at least, no participants required this treatment [12].

Smoking cessation and prevention of respiratory complications through postoperative chest therapy were included in the intervention of two studies [10, 12]. Psychological support addressing fatigue, anxiety and depression in the perioperative period was included in only one intervention [10]. Optimisation of chronic medical conditions, primarily through prescribing or deprescribing medications, was addressed by only one intervention [11].

Medically trained staff, including surgeons, anaesthetists and geriatricians, were involved in delivering the intervention in three studies [11, 12, 14], and in two studies [10, 13], they were supported by a multidisciplinary team comprising nurses and allied health professionals. In the geriatrician-led intervention study [11], multidisciplinary team members were only available in the postoperative inpatient setting.

### Outcomes

**Fig. 2:**
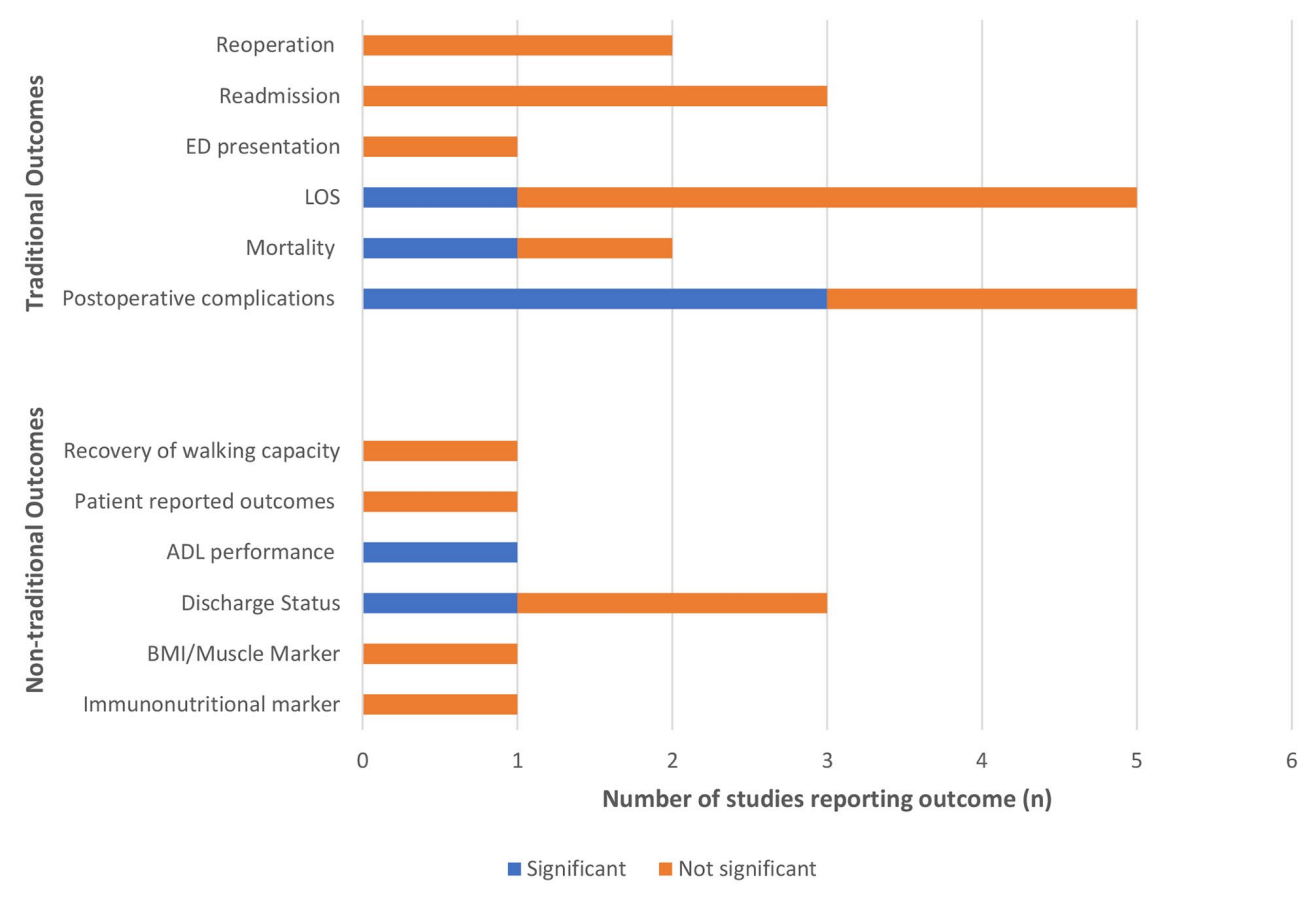
Statistically significant (blue) and non-statistically significant (orange) outcomes of perioperative multicomponent interventions. Note: ADLs, activities of daily living; ED, emergency department; BMI, Body Mass Index. Patient reported outcomes included health status, anxiety and depression, energy expenditure. Discharge status referred to change in function + discharge disposition, new institutionalisation, or discharge home. Immunonutritional markers included neutrophil lymphocyte ratio (NLR), lymphocyte to C-reactive protein ratio (LCR), prognostic nutritional index (PNI), albumin; Muscle markers included soft lean mass, and skeletal muscle mass

All studies measured multiple traditional surgical outcomes (Table 2). All five studies reported on length of stay and postoperative complications. The effectiveness of the interventions on these outcomes was mixed (Fig. 2). In Mazzola et al.’s study [12], mortality at 30 days and 3 months was significantly lower in the intervention group than the control group in univariate analyses (zero versus 14%, *p* = 0.01; zero versus 28%, *p* < 0.001). Overall and severe complications were significantly lower in the intervention group than the control group (41% versus 74%, *p* = 0.005; 17% versus 43%, *p* = 0.02) [12]. Similarly, in Suzuki et al.’s [14] study, univariate analyses showed that rates of severe, multiple complications were significantly lower in the intervention group than the control group (6.7% versus 21.2%, *p* = 0.04). The adjusted odds ratio for complications was 0.33 (95% CI = 0.11–0.95) in Ommundsen et al.’s [11] study. Wada et al.’s [13] study was the only one to report a statistically significant differences in length of stay between the intervention and control groups (13.0 days versus 15.9 days, *p* = 0.03).

All studies measured one or more non-traditional outcome. Patient-centred outcomes included recovery of walking capacity, patient-reported health status, anxiety and depression and energy expenditure [10], discharge status [11, 12, 14] (including new institutionalisation) [12, 14] and ADL performance [14]. One study included a range of physical outcomes, including measures of nutritional status and physical parameters [13]. There was some evidence for a significant effect of multicomponent intervention on patient-centred outcomes (Fig. 2). In Suzuki et al.’s [14] study, ADL deterioration was significantly lower in the intervention group than the control group (6.7% versus 21.1%, *p* = 0.04; 6.7% versus 39.5%, *p* = 0.02) and those in the intervention group were more likely to be independent and living at home postoperatively (80.0% versus 60.5%, *p* = 0.02).

### Risk of bias

**Fig. 3:**
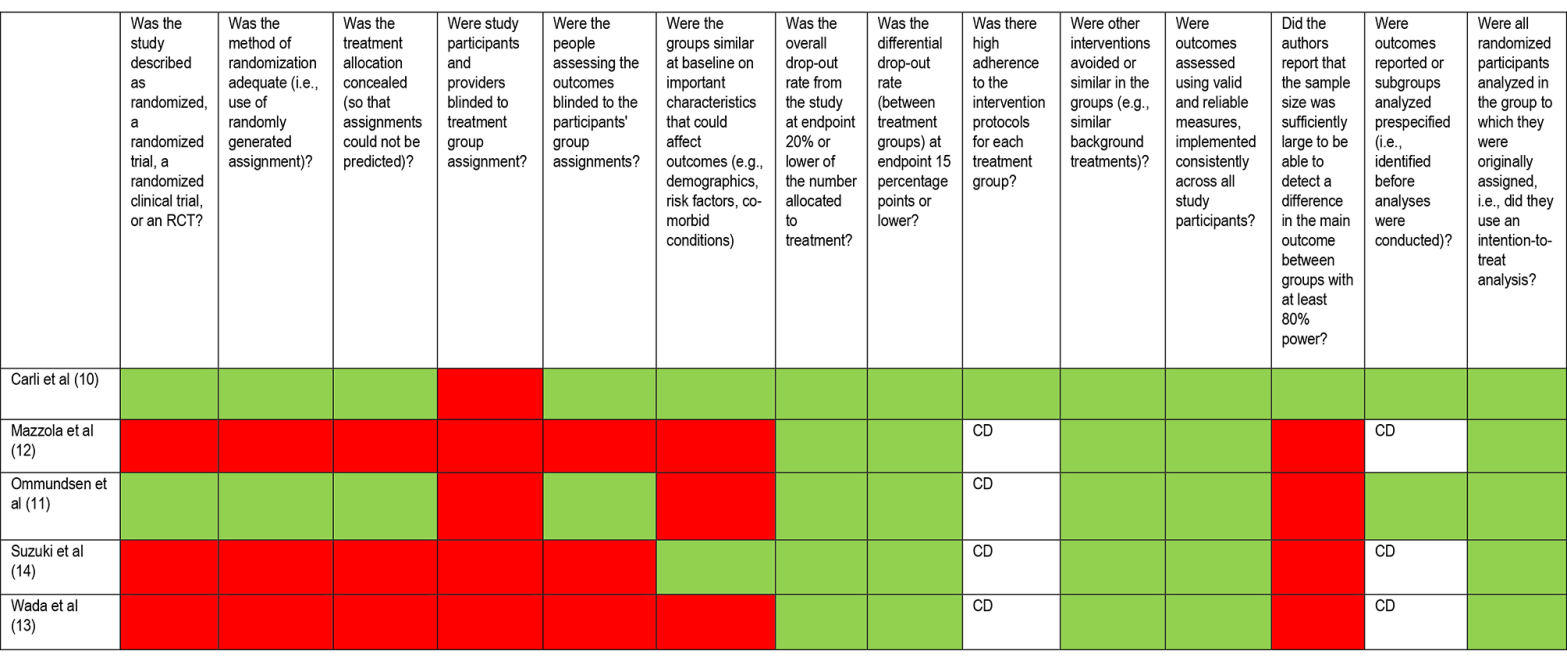
Risk of bias assessment using the National Institute of Health Quality Tool [9]. Note: Green represents low risk of bias, and red high risk of bias. For domains designated CD, risk of bis was unclear / could not be determined

The assessment of risk of bias for included studies are summarised in Fig. 3. Risk of bias arose primarily due to lack of randomization [12–14] and blinding [10–14]. Due to the nature of the intervention, it was not possibly for participants or intervention staff to be blinded. However, only two studies blinded outcome assessors to the participants’ group assignments [10, 11]. Only one study reported sample size and power analysis [10]. Overall, the quality of the evidence was rated as fair.

## Discussion

Our systematic review of literature published since 2016 identified five studies of perioperative multicomponent interventions in frail patients undergoing elective oncologic gastrointestinal surgery. There were two RCTs and three quasi-experimental study designs and overall, the quality of the evidence was deemed to be fair. The studies did not consistently demonstrate improvements in outcomes. Reductions in postoperative complications, mortality, length of stay and functional deterioration were reported yet methodological issues and substantial heterogeneity of the interventions precludes drawing clear conclusions regarding the optimal model of care.

In 2017, McIsaac et al. [7] also found that studies of multicomponent interventions did not consistently demonstrate improvements in outcomes. They attributed this, in part, to poor adherence and protocol implementation issues [7]. Certainly, there is evidence for a dose-response relationship between ERAS protocol adherence and clinical outcomes after major colorectal surgery [15] and it is probably reasonable to expect a similar effect with other perioperative interventions. Protocol adherence was identified as an issue in two studies [10, 11] included in our review. The interventions in these two studies were embedded within a well-established ERAS program and, interestingly, the authors speculated that the study interventions may have had a limited effect, especially with respect to surgical outcomes, given that other aspects of perioperative care were optimised [10, 11].

The sample sizes of included studies were modest – only one study was adequately powered for the primary outcomes (and found no significant difference between the intervention and control groups for any outcome) [10]. The quasi-experimental studies [12–14] were retrospective and there were important differences in baseline characteristics in both quasi-experimental [12, 13] and RCT studies [11]. Statistical analyses were largely unadjusted, failing to account for potential confounding factors. For example, a statistically significant difference in mortality rates in intervention and control groups in Mazzola et al.’s [12] study may have been confounded by differences in the rates of pancreatic cancer, a malignancy associated with an extremely poor prognosis [16].

There was substantial variability among the interventions tested and, as such, it is difficult to ascertain which elements are the key ingredients for an effective intervention in this setting and patient population. All studies addressed physical activity and nutrition, which is in keeping with ERAS guidelines for elective colorectal surgical patients [17]. Nutrition and physical activity interventions, addressing protein/caloric supplementation and resistance-based training, respectively, are also recommended for the management of frailty more generally [18]. Health domains known to be relevant to the care of frail adults, such as social support, was not addressed by the interventions in the included studies and only one study [11] included a review of medical co-morbidity and medications in their intervention.

Comprehensive Geriatric Assessment (CGA) is a comprehensive evaluation by a medical specialist with expertise in geriatric medicine to identify and address medical, social and functional needs, optimise medication prescribing, and engage a multidisciplinary team to assist frail patients to attain goals [19]. It is, by definition, a multicomponent intervention. Recommended by Best Practice Guidelines as the approach to managing frailty in *all* patients [20], CGA has been shown to increase the likelihood of frail inpatients being alive and in their own homes at follow-up [21]. The study by Ommundsen et al. [11], which we included in our systematic review, described an intervention including a geriatric assessment and tailored management plan. This intervention appears to align with the principles of CGA; however, the authors of the study noted that there was minimal access to multidisciplinary allied health input and the time between assessment and surgery was very short (median = 6 days). The intervention did not appear to reduce the rates of traditional adverse surgical outcomes in this relatively small study, which is consistent with meta-analysis of data from studies of preoperative CGA in elective non-cardiac high-risk surgery [22]. Even so, preoperative CGA is recommended in recent clinical practice guidelines for the perioperative care of frail people undergoing surgery [23].

Three studies included in our review were ‘prehabilitation’ studies implementing interventions between five days and four weeks prior to surgery [10, 12, 13]. Prehabilitation is designed to improve an individual’s resilience prior to elective surgery [24]. The evidence suggests that preoperative interventions need to be implemented a reasonably long time, at least four weeks, prior to surgery in order to build physiological reserve [25]. However, this is not always feasible. In cancer surgery, for example, delays in treatment can result in poor oncological outcomes. Neoadjuvant therapy increases the time from diagnosis until surgery [26] and is associated with a decrease in overall physical fitness, which has been associated with worse outcomes after surgery [27, 28]. Variations in the timing of intervention likely contributes to variability of the study results reported here. Notably, due to there being “no evidence that prehabilitation programmes improve postoperative outcomes for older patients or those living with frailty” (p. 3), current guidelines advocate the use of CGA [23].

The intervention evaluated by Suzuki and colleagues [14] primarily occurred in the postoperative period. While it was not a CGA, the intervention addressed multiple health domains with the support of a multidisciplinary team of medical and allied health professionals. Although it was a small study, it demonstrated significantly lower complications and dependence in the intervention group. Compared with the other studies included in this systematic review, the results of Suzuki et al.’s [14] study may be more generalisable to *emergent* surgical populations who are able to receive postoperative (and not preoperative) interventions. CGA conducted postoperatively in hip fracture patients, for example, has been shown to reduce the risk of mortality, readmission and new institutionalisation [29].

Heterogeneity of outcome measures was identified by McIsaac and colleagues [7] as a key issue in their systematic review. In our review, effectiveness was primarily measured using a variety of traditional surgical outcomes. It is possible, however, that perioperative interventions in frail surgical patients will have minor effects on traditional outcomes and major effects on patient-centred outcomes such as functional decline, quality of life and discharge disposition. In our systematic review, four studies reported on patient-centred outcomes. None of the studies examined effects of intervention on delirium or cognitive decline, which along with functional decline, are increasingly prioritised by older patients and are of critical importance to informed surgical decision-making [30]. Furthermore, despite measuring frailty at baseline, none of the studies examined changes in frailty status following multicomponent interventions as an outcome measure. Patient-centred outcomes are not commonly evaluated in clinical trials of frail patients in hospital [31]. The evidence suggests that many patients with severe illness would not elect for life-sustaining treatment if the burden of treatment was high or if treatment resulted in significant cognitive and functional impairment [32]. We agree with McIsaac and colleagues that ascertaining what outcomes are most important to frail patients and the people who care for them is necessary to inform future clinical trials. This is a focus of ongoing work by our group.

### Strengths and limitations

This systematic review used a comprehensive search strategy with defined inclusion and exclusion criteria, which was broad enough to encompass all types of surgery, in elective and emergent settings, yet narrow enough to permit a synthesis of evidence relating to a particular population group and type of intervention. This review therefore provided important insights into the current state of evidence of the effectiveness of multicomponent perioperative interventions in frail surgical patients.

There are limitations to this study. Despite the broad search strategy, all included studies were conducted in elective oncologic gastrointestinal surgery populations, limiting generalisability of results. The small number of included studies may reflect our protocol’s requirement that a validated measure of frailty be used and that the majority of the study sample be classified as frail. This resulted in exclusion of studies of multicomponent interventions in what may be generally accepted to be frail patient populations, such as orthogeriatric models of care in hip fracture. Nevertheless, as it is well-understood that frail surgical patients are clinically different to non-frail surgical patients and clinical practice guidelines emphasise the importance of using validated tools to diagnose frailty in surgical patients, only including studies that used a validated frailty measure ensured that the evidence presented here is clinically relevant.

## Conclusion

The findings of this systematic review mirror those of McIsaac et al.’s [7] review – relatively few studies of perioperative multicomponent interventions in frail patients have been conducted over the last seven years and there is variability in outcomes. We conclude that perioperative multicomponent interventions, some of which align more closely with ‘prehabilitation’ and others with CGA, *may* improve some traditional surgical and patient-centred outcomes in frail older adults undergoing elective oncologic gastrointestinal surgery. However, more low-risk of bias studies are needed to determine the effectiveness of interventions in samples of frail adults undergoing other types of surgery and implementation studies are needed to tease apart the critical elements of interventions and to identify enablers and barriers to protocol adherence. Attention must also turn to ascertaining what outcomes are most valued by frail surgical patients and the people who care for them. Incorporating these outcomes into future clinical trials will make comparisons between trials easier and will assist patients, clinicians and policy-makers to make more informed management decisions.

### Electronic supplementary material

Below is the link to the electronic supplementary material.

Supplementary Material 1

## Data Availability

All data generated or analysed during this study are included in this published article and its supplementary information files.

## Abbreviations

CINAHL: Cumulative Index to Nursing and Allied Health Literature
EMBASE: Excerpta Medica Database
LOS: Length of stay
PROSPERO: International prospective register of systematic reviews
MESH: Medical subject headings
PRISMA: Preferred Reporting Items for Systematic Reviews
CGA: Comprehensive geriatric assessment
ERAS: Enhanced Recovery After Surgery
mFI: Modified Frailty Index
mFI-11: Modified Frailty Index-11
CFS: Clinical Frailty Scale
VES-13: Vulnerable Elders Survey
LOS: Length of stay
ED: Emergency department
TPN: Total parenteral nutrition
ADLs: Activities of daily living
CRP: C-reactive protein
NJ: Nasojejunal
PN: Parenteral nutrition
CD: Cannot Determine
POMT: Perioperative Management Team
RCT: Randomised controlled trial
NLR: Neutrophil lymphocyte ratio
LCR: Lymphocyte to C-reactive protein ratio
PNI: Prognostic nutritional index
