# Supplementary material for: Multicomponent perioperative interventions to improve outcomes for frail patients: a systematic review
Source: BMC Geriatr. 2024 Apr 26;24:376. doi: 10.1186/s12877-024-04985-4 (PMC11055226; doi:10.1186/s12877-024-04985-4)
Supplement: Supplementary file 1 — Supplementary Material 1 [file 12877_2024_4985_MOESM1_ESM.docx]

**SUPPLEMENTARY DATA**

**Search strategies for each included database**

**Pubmed**

#1. Frail Elderly[MeSH Major Topic]

#2. frail*[Title]

#3. #1 OR #2

#4. Perioperative Period[MeSH Major Topic] OR Surgical Procedures, Operative[MeSH Major Topic] OR Elective Surgical Procedures[MeSH Major Topic]

#5. perioperative*[Title] OR peri-operative*[Title] OR preoperative*[Title] OR pre-operative*[Title] OR postoperative*[Title] OR post-operative*[Title] OR surgery[Title] OR surgical[Title]

#6. #4 OR #5

#7. #3 AND #6

**Embase**

#1 ’frail elderly’/exp/mj

#2 ‘frailty':ti

#3 #1 OR #2

#4 'perioperative period'/exp/mj OR 'surgery'/exp/mj OR 'elective surgical procedures’/exp/mj

#5 perioperative*:ti OR 'peri operative*':ti OR preoperative*:ti OR 'pre operative*':ti OR postoperative*:ti OR 'post operative*':ti OR surgery:ti OR surgical:ti

#6 #4 OR #5

#7 #3 AND #6

#8 #7 AND (**2022**:py)

**Cochrane**

#1 [mh "Frail Elderly" [mj]]

#2 Frail*:ti

#3 #1 OR #2

#4 [mh "Perioperative Period" [mj]] OR [mh "Surgical Procedures, Operative" [mj]] OR [mh "Elective Surgical Procedures" [mj]]

#5 perioperative*:ti OR peri-operative*:ti OR preoperative*:ti OR pre-operative*:ti OR postoperative*:ti OR post-operative*:ti OR surgery:ti OR surgical:ti

#6 #4 OR 5

#7 #3 AND #6

**CINAHL**

S1 MM Frail Elderly+

S2 TI frail*

S3 S1 OR S2

S4 MM Perioperative Care+ OR MM Surgery, Operative+ OR MM Surgery, Elective+

S5 TI perioperative* OR peri-operative* OR TI preoperative* OR TI pre-operative* OR TI postoperative* OR TI post-operative* OR TI surgery OR TI surgical

S6 S4 OR S5

S7 S3 AND S6
